# 2-Fluoro-*N*′-(2-meth­oxy­benzyl­idene)benzohydrazide

**DOI:** 10.1107/S1600536810050853

**Published:** 2010-12-11

**Authors:** Cheng-Bi Xu, Zong-Gui Wang, Yi Nan, Ling Yuan, Rong Wang, Shu-Xiang Zhang

**Affiliations:** a The Second Hospital of Jilin University, Changchun Jilin 130041, People’s Republic of China; b Traditional Chinese Medicine College of Ningxia Medical University, Yinchuan Ningxia 750004, People’s Republic of China; c Pharmacy College of Ningxia Medical University, Yinchuan Ningxia 750004, People’s Republic of China; d Minority Traditional Medical Center of Minzu University of China, Beijing 100081, People’s Republic of China; e Affiliated Hospital of Ningxia Medical University, Yinchuan Ningxia 750004, People’s Republic of China

## Abstract

The mol­ecule of the title compound, C_15_H_13_FN_2_O_2_, exists in a *trans* configuration with respect to the methyl­idene unit. The two benzene rings form a dihedral angle of of 64.7 (2)°. In the crystal, mol­ecules are linked through N—H⋯O hydrogen bonds into chains propagating along the *c* axis.

## Related literature

For the reference bond lengths, see: Allen *et al.* (1987 ▶). For structural studies of hydrazone compounds, see: Han & Zhao (2010 ▶); Zhou & Yang (2010 ▶); Huang & Wu (2010 ▶); Shalash *et al.* (2010 ▶). For a related structure, see: Xu *et al.* (2011 ▶).
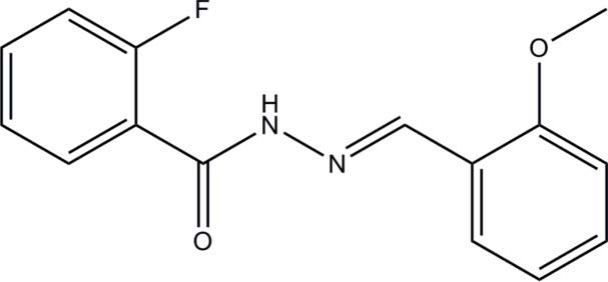


## Experimental

### 

#### Crystal data

C_15_H_13_FN_2_O_2_
*M* *_r_* = 272.27
Monoclinic, 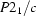

*a* = 13.612 (2) Å
*b* = 12.278 (2) Å
*c* = 7.925 (1) Å
β = 92.802 (3)°
*V* = 1322.9 (3) Å^3^
*Z* = 4
Mo *K*α radiation
μ = 0.10 mm^−1^
*T* = 298 K
0.17 × 0.13 × 0.12 mm

#### Data collection

Bruker SMART CCD area-detector diffractometer
Absorption correction: multi-scan (*SADABS*; Sheldrick, 1996 ▶) *T*_min_ = 0.983, *T*_max_ = 0.988
10047 measured reflections
2821 independent reflections
1643 reflections with *I* > 2σ(*I*)
*R*_int_ = 0.047

#### Refinement

*R*[*F* ^2^ > 2σ(*F* ^2^)] = 0.058
*wR*(*F* ^2^) = 0.164
*S* = 1.03
2821 reflections
182 parameters
H-atom parameters constrained
Δρ_max_ = 0.47 e Å^−3^
Δρ_min_ = −0.26 e Å^−3^

### 

Data collection: *SMART* (Bruker, 1998 ▶); cell refinement: *SAINT* (Bruker, 1998 ▶); data reduction: *SAINT*; program(s) used to solve structure: *SHELXS97* (Sheldrick, 2008 ▶); program(s) used to refine structure: *SHELXL97* (Sheldrick, 2008 ▶); molecular graphics: *SHELXTL* (Sheldrick, 2008 ▶); software used to prepare material for publication: *SHELXTL*.

## Supplementary Material

Crystal structure: contains datablocks global, I. DOI: 10.1107/S1600536810050853/cv5011sup1.cif

Structure factors: contains datablocks I. DOI: 10.1107/S1600536810050853/cv5011Isup2.hkl

Additional supplementary materials:  crystallographic information; 3D view; checkCIF report

## Figures and Tables

**Table 1:** Hydrogen-bond geometry (Å, °)

*D*—H⋯*A*	*D*—H	H⋯*A*	*D*⋯*A*	*D*—H⋯*A*
N1—H1⋯O1^i^	0.86	2.08	2.866 (2)	152

Symmetry code: (i) 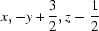.

**Fig. 1.**
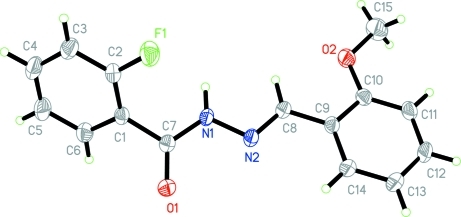
The molecular structure of the title compound, with displacement ellipsoids drawn at the 30% probability level.

**Fig. 2.**
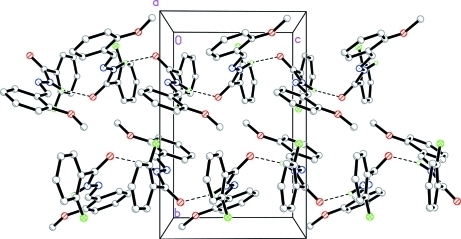
A portion of the crystal packing showing the hydrogen-bonded (dashed lines) chains of the molecules. H atoms omitted for clarity/

## supplementary crystallographic information

### Comment

As a contribution to a structural study of hydrazone compounds (Han & Zhao, 2010; Zhou & Yang, 2010; Huang & Wu, 2010; Shalash *et al.*, 2010), we present here the crystal structure of the title compound.

The molecule of the title compound exists in a *trans* configuration with respect to the methylidene unit (Fig. 1). The molecule is twisted, with the dihedral angle between the two benzene rings of 64.7 (2)°. The torsion angle C7—N1—N2—C8 is 8.6 (2)°. The bond lengths are within normal ranges (Allen *et al.*, 1987) and are comparable with those observed in the similar compound (Xu *et al.*, 2011).

In the crystal structure, molecules are linked through intermolecular N—H···O hydrogen bonds (Table 1), to form chains down the *c* axis (Fig. 2).

### Experimental

2-Methoxybenzaldehyde (0.1 mmol, 13.6 mg) and 2-fluorobenzohydrazide (0.1 mmol, 15.4 mg) were mixed in ethanol (20 ml). The mixture was stirred at room temperature to give a clear colorless solution. Colourless well shaped crystals of the title compound were formed by gradual evaporation of the solvent over a period of five days at room temperature.

### Refinement

All H atoms were placed in geometrically idealized positions, with C—H = 0.93-0.96 Å, N—H = 0.86 Å, and refined as riding, with U_iso_(H) = 1.2U_eq_(C,N) and 1.5U_eq_(C15).

### Crystal data

**Table:**

C_15_H_13_FN_2_O_2_	*F*(000) = 568
*M**_r_* = 272.27	*D*_x_ = 1.367 Mg m^−^^3^
Monoclinic, *P*2_1_/*c*	Mo *K*α radiation, λ = 0.71073 Å
Hall symbol: -P 2ybc	Cell parameters from 1625 reflections
*a* = 13.612 (2) Å	θ = 2.2–26.4°
*b* = 12.278 (2) Å	µ = 0.10 mm^−^^1^
*c* = 7.925 (1) Å	*T* = 298 K
β = 92.802 (3)°	Block, colorless
*V* = 1322.9 (3) Å^3^	0.17 × 0.13 × 0.12 mm
*Z* = 4	

### Data collection

**Table:**

Bruker SMART CCD area-detector diffractometer	2821 independent reflections
Radiation source: fine-focus sealed tube	1643 reflections with *I* > 2σ(*I*)
graphite	*R*_int_ = 0.047
ω scans	θ_max_ = 27.0°, θ_min_ = 1.5°
Absorption correction: multi-scan (*SADABS*; Sheldrick, 1996)	*h* = −17→16
*T*_min_ = 0.983, *T*_max_ = 0.988	*k* = −15→15
10047 measured reflections	*l* = −10→10

### Refinement

**Table:**

Refinement on *F*^2^	Primary atom site location: structure-invariant direct methods
Least-squares matrix: full	Secondary atom site location: difference Fourier map
*R*[*F*^2^ > 2σ(*F*^2^)] = 0.058	Hydrogen site location: inferred from neighbouring sites
*wR*(*F*^2^) = 0.164	H-atom parameters constrained
*S* = 1.03	*w* = 1/[σ^2^(*F*_o_^2^) + (0.0839*P*)^2^] where *P* = (*F*_o_^2^ + 2*F*_c_^2^)/3
2821 reflections	(Δ/σ)_max_ < 0.001
182 parameters	Δρ_max_ = 0.47 e Å^−^^3^
0 restraints	Δρ_min_ = −0.26 e Å^−^^3^

### Special details

**Table:**

Geometry. All esds (except the esd in the dihedral angle between two l.s. planes) are estimated using the full covariance matrix. The cell esds are taken into account individually in the estimation of esds in distances, angles and torsion angles; correlations between esds in cell parameters are only used when they are defined by crystal symmetry. An approximate (isotropic) treatment of cell esds is used for estimating esds involving l.s. planes.
Refinement. Refinement of F^2^ against ALL reflections. The weighted R-factor wR and goodness of fit S are based on F^2^, conventional R-factors R are based on F, with F set to zero for negative F^2^. The threshold expression of F^2^ > 2sigma(F^2^) is used only for calculating R-factors(gt) etc. and is not relevant to the choice of reflections for refinement. R-factors based on F^2^ are statistically about twice as large as those based on F, and R- factors based on ALL data will be even larger.

### Fractional atomic coordinates and isotropic or equivalent isotropic displacement parameters (Å^2^)

**Table:**

	*x*	*y*	*z*	*U*_iso_*/*U*_eq_	
F1	0.89639 (10)	0.58503 (11)	0.9614 (2)	0.0657 (5)	
N1	0.72769 (12)	0.71726 (15)	0.9490 (2)	0.0409 (5)	
H1	0.7427	0.6742	0.8687	0.049*	
N2	0.63391 (12)	0.71379 (15)	1.0089 (2)	0.0392 (5)	
O1	0.77942 (11)	0.85629 (12)	1.1218 (2)	0.0442 (4)	
O2	0.43863 (11)	0.55230 (14)	0.7104 (2)	0.0561 (5)	
C1	0.89489 (15)	0.77601 (18)	0.9435 (3)	0.0369 (5)	
C2	0.94195 (16)	0.67840 (18)	0.9204 (3)	0.0414 (6)	
C3	1.03448 (17)	0.6706 (2)	0.8625 (3)	0.0546 (7)	
H3	1.0642	0.6030	0.8504	0.065*	
C4	1.08295 (18)	0.7651 (3)	0.8224 (4)	0.0603 (8)	
H4	1.1458	0.7614	0.7817	0.072*	
C5	1.03917 (19)	0.8636 (2)	0.8423 (4)	0.0615 (8)	
H5	1.0721	0.9269	0.8143	0.074*	
C6	0.94636 (17)	0.8701 (2)	0.9034 (3)	0.0508 (7)	
H6	0.9177	0.9379	0.9182	0.061*	
C7	0.79557 (15)	0.78675 (18)	1.0143 (3)	0.0367 (6)	
C8	0.57425 (15)	0.65389 (18)	0.9208 (3)	0.0384 (6)	
H8	0.5954	0.6201	0.8240	0.046*	
C9	0.47321 (15)	0.63784 (17)	0.9704 (3)	0.0376 (6)	
C10	0.40509 (16)	0.58424 (17)	0.8620 (3)	0.0418 (6)	
C11	0.31043 (16)	0.56584 (19)	0.9121 (4)	0.0504 (7)	
H11	0.2652	0.5297	0.8403	0.061*	
C12	0.28342 (17)	0.6008 (2)	1.0670 (4)	0.0591 (8)	
H12	0.2197	0.5881	1.0996	0.071*	
C13	0.34873 (18)	0.6544 (2)	1.1753 (4)	0.0607 (8)	
H13	0.3295	0.6781	1.2801	0.073*	
C14	0.44315 (17)	0.6725 (2)	1.1266 (3)	0.0500 (7)	
H14	0.4876	0.7086	1.1998	0.060*	
C15	0.3743 (2)	0.4906 (3)	0.6007 (4)	0.0838 (10)	
H15A	0.3546	0.4257	0.6575	0.126*	
H15B	0.4076	0.4711	0.5010	0.126*	
H15C	0.3172	0.5333	0.5694	0.126*	

### Atomic displacement parameters (Å^2^)

**Table:**

	*U*^11^	*U*^22^	*U*^33^	*U*^12^	*U*^13^	*U*^23^
F1	0.0498 (9)	0.0509 (9)	0.0966 (13)	−0.0034 (7)	0.0045 (8)	0.0114 (9)
N1	0.0280 (10)	0.0526 (12)	0.0425 (12)	−0.0043 (8)	0.0072 (9)	−0.0084 (9)
N2	0.0299 (10)	0.0461 (11)	0.0420 (12)	−0.0021 (8)	0.0045 (9)	0.0006 (9)
O1	0.0435 (9)	0.0482 (9)	0.0412 (10)	−0.0005 (7)	0.0046 (8)	−0.0040 (8)
O2	0.0504 (10)	0.0698 (12)	0.0480 (11)	−0.0142 (9)	0.0016 (9)	−0.0147 (9)
C1	0.0306 (11)	0.0478 (13)	0.0323 (13)	−0.0031 (10)	0.0004 (10)	0.0015 (11)
C2	0.0331 (13)	0.0460 (14)	0.0449 (15)	−0.0051 (11)	−0.0007 (11)	0.0034 (12)
C3	0.0377 (14)	0.0665 (17)	0.0593 (18)	0.0072 (12)	0.0019 (13)	−0.0005 (14)
C4	0.0358 (14)	0.083 (2)	0.0631 (19)	−0.0034 (14)	0.0111 (13)	0.0023 (16)
C5	0.0482 (16)	0.0693 (19)	0.068 (2)	−0.0163 (14)	0.0163 (14)	0.0086 (16)
C6	0.0441 (14)	0.0538 (15)	0.0550 (17)	−0.0073 (12)	0.0078 (13)	0.0024 (13)
C7	0.0349 (12)	0.0434 (13)	0.0319 (13)	−0.0011 (10)	0.0016 (10)	0.0050 (11)
C8	0.0322 (12)	0.0444 (13)	0.0387 (14)	0.0013 (10)	0.0029 (10)	0.0020 (11)
C9	0.0313 (12)	0.0347 (12)	0.0470 (15)	0.0000 (9)	0.0021 (11)	0.0021 (11)
C10	0.0393 (13)	0.0362 (13)	0.0497 (16)	−0.0007 (10)	0.0009 (12)	−0.0001 (11)
C11	0.0333 (13)	0.0487 (15)	0.069 (2)	−0.0054 (11)	−0.0012 (13)	−0.0069 (13)
C12	0.0325 (13)	0.0613 (16)	0.085 (2)	−0.0047 (12)	0.0172 (14)	−0.0060 (16)
C13	0.0435 (15)	0.0712 (18)	0.069 (2)	−0.0037 (13)	0.0179 (14)	−0.0168 (16)
C14	0.0390 (14)	0.0585 (16)	0.0531 (17)	−0.0051 (11)	0.0087 (12)	−0.0129 (13)
C15	0.080 (2)	0.112 (3)	0.059 (2)	−0.0263 (19)	−0.0072 (17)	−0.0299 (19)

### Geometric parameters (Å, °)

**Table:**

F1—C2	1.351 (2)	C5—H5	0.9300
N1—C7	1.343 (3)	C6—H6	0.9300
N1—N2	1.384 (2)	C8—C9	1.462 (3)
N1—H1	0.8600	C8—H8	0.9300
N2—C8	1.278 (3)	C9—C14	1.390 (3)
O1—C7	1.234 (3)	C9—C10	1.397 (3)
O2—C10	1.364 (3)	C10—C11	1.385 (3)
O2—C15	1.422 (3)	C11—C12	1.368 (4)
C1—C2	1.375 (3)	C11—H11	0.9300
C1—C6	1.395 (3)	C12—C13	1.373 (4)
C1—C7	1.495 (3)	C12—H12	0.9300
C2—C3	1.365 (3)	C13—C14	1.378 (3)
C3—C4	1.379 (4)	C13—H13	0.9300
C3—H3	0.9300	C14—H14	0.9300
C4—C5	1.361 (4)	C15—H15A	0.9600
C4—H4	0.9300	C15—H15B	0.9600
C5—C6	1.377 (3)	C15—H15C	0.9600
			
C7—N1—N2	121.09 (19)	N2—C8—H8	119.6
C7—N1—H1	119.5	C9—C8—H8	119.6
N2—N1—H1	119.5	C14—C9—C10	118.4 (2)
C8—N2—N1	113.74 (19)	C14—C9—C8	121.3 (2)
C10—O2—C15	117.99 (19)	C10—C9—C8	120.2 (2)
C2—C1—C6	116.6 (2)	O2—C10—C11	124.2 (2)
C2—C1—C7	124.2 (2)	O2—C10—C9	115.86 (19)
C6—C1—C7	119.1 (2)	C11—C10—C9	120.0 (2)
F1—C2—C3	117.6 (2)	C12—C11—C10	120.0 (2)
F1—C2—C1	119.0 (2)	C12—C11—H11	120.0
C3—C2—C1	123.3 (2)	C10—C11—H11	120.0
C2—C3—C4	118.6 (2)	C11—C12—C13	121.1 (2)
C2—C3—H3	120.7	C11—C12—H12	119.4
C4—C3—H3	120.7	C13—C12—H12	119.4
C5—C4—C3	120.3 (2)	C12—C13—C14	119.1 (3)
C5—C4—H4	119.9	C12—C13—H13	120.4
C3—C4—H4	119.9	C14—C13—H13	120.4
C4—C5—C6	120.4 (2)	C13—C14—C9	121.3 (2)
C4—C5—H5	119.8	C13—C14—H14	119.4
C6—C5—H5	119.8	C9—C14—H14	119.4
C5—C6—C1	120.8 (2)	O2—C15—H15A	109.5
C5—C6—H6	119.6	O2—C15—H15B	109.5
C1—C6—H6	119.6	H15A—C15—H15B	109.5
O1—C7—N1	124.29 (19)	O2—C15—H15C	109.5
O1—C7—C1	121.0 (2)	H15A—C15—H15C	109.5
N1—C7—C1	114.7 (2)	H15B—C15—H15C	109.5
N2—C8—C9	120.8 (2)		

### Hydrogen-bond geometry (Å, °)

**Table:**

*D*—H···*A*	*D*—H	H···*A*	*D*···*A*	*D*—H···*A*
N1—H1···O1^i^	0.86	2.08	2.866 (2)	152.

Symmetry codes: (i) *x*, −*y*+3/2, *z*−1/2.
